# Multifactor Risk Stratification for Post‐Transplant Alcohol Relapse Using Abstinence, Psychosocial, and Socioeconomic Factors

**DOI:** 10.1002/ags3.70193

**Published:** 2026-02-15

**Authors:** Ayato Obana, Garren Montgomery, Miho Akabane, Matthew Hamilton, Navdeep Singh, Musab Alebrahim, Austin Schenk, Sylvester Black, Kenneth Washburn, Khalid Mumtaz

**Affiliations:** ^1^ Comprehensive Transplant Center, Department of Surgery Ohio State University Wexner Medical Center Ohio USA; ^2^ Department of Hepato‐Biliary‐Pancreatic and Gastrointestinal Surgery, School of Medicine International University of Health and Welfare Chiba Japan; ^3^ Division of Gastroenterology, Hepatology and Nutrition Ohio State University, Wexner Medical Center Ohio USA

**Keywords:** alcohol relapse, alcohol‐related liver disease, liver transplantation, risk assessment, socioeconomic factor

## Abstract

**Aim:**

Post‐liver transplant (LT) alcohol relapse complicates long‐term outcomes and organ allocation. The traditional “six‐month abstinence rule” remains widely used, but relapse risk is also shaped by psychosocial, socioeconomic, and psychiatric factors. We examined the impact of pre‐LT abstinence duration on post‐LT alcohol relapse and developed a multivariable risk model integrating abstinence, psychosocial assessment, and socioeconomic status.

**Methods:**

In this single‐center retrospective study, 383 adults undergoing LT for alcohol‐related liver disease were included.

**Results:**

Any post‐LT alcohol relapse occurred in 20.9% (*n* = 80). Relapse phenotypes were non‐mutually exclusive: sustained alcohol use (77.5%), harmful drinking (68.8%), and recurrent ALD (46.3%). On multivariable analysis, shorter pre‐LT abstinence, higher Stanford Integrated Psychosocial Assessment for Transplantation (SIPAT) scores, and higher educational attainment were independently associated with relapse. Each additional month of abstinence reduced relapse odds by 4%, whereas each one‐point increase in SIPAT increased relapse odds by 3%. Cumulative risk curves demonstrated a non‐linear relationship between abstinence duration and relapse, with risk peaking around 9 months and declining thereafter across all relapse phenotypes. A prediction model combining abstinence duration, SIPAT score, and education achieved an area under the receiver operating characteristic curve (AUC) of 0.70, with consistent performance on fivefold cross‐validation.

**Conclusion:**

These findings support a multifactorial approach to relapse risk stratification that goes beyond a fixed 6‐month abstinence rule. Incorporating abstinence duration together with structured psychosocial assessment and education level may better inform both transplant listing decisions and the intensity of post‐LT addiction care for patients.

## Graphical abstract

Alcohol relapse after liver transplantation is difficult to predict using abstinence duration alone. We developed a multifactor model integrating abstinence duration, psychosocial risk (SIPAT), and socioeconomic context (AUC 0.70). This approach may support individualized risk assessment and tailored follow‐up intensity; external validation is needed.

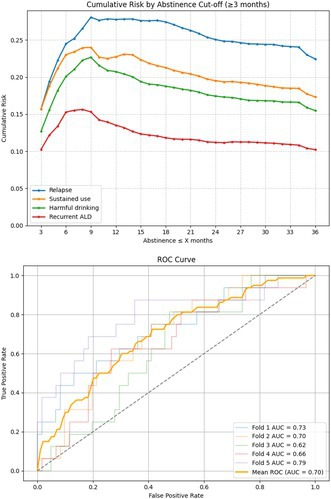

**Abbreviations**
AA: Alcoholics Anonymous
AAH: Acute alcoholic‐associated hepatitis
ACLF: Acute‐on‐chronic liver failure
ALD: Alcohol‐related Liver Disease
AOD: Alcohol and Other Drugs
AUD: Alcohol Use Disorder
BMI: Body Mass Index
DUI: Driving Under the Influence
HRAR: High‐Risk Alcoholism Relapse
LT: Liver Transplantation
LTR: Liver Transplant Recipient
MELD‐Na: Model for End‐Stage Liver Disease‐Sodium
NIAAA: National Institute on Alcohol Abuse and Alcoholism
OSUWMC: Ohio State University Wexner Medical Center
PEth: Phosphatidylethanol
SALT: Sustained Alcohol use Post‐Liver Transplant
SIPAT: Stanford Integrated Psychosocial Assessment for Transplantation

## 1 Introduction

Alcohol‐related liver disease (ALD) is a leading indication for liver transplantation (LT) worldwide, with proportionately increasing trends over recent years [1]. Although LT improves survival, alcohol relapse after LT is common [2] and is associated with graft dysfunction [3], chronic rejection [4], and death [4]. Prior studies report relapse in roughly one fifth of recipients within several years [5], although estimates vary depending on the definition of relapse and ascertainment methods [5]. Accurately predicting relapse risk and selecting appropriate candidates is critically important for LT recipients outcomes and effective utilization of limited organ resources [6].

Historically, many programs have relied on a “six‐month abstinence rule” as a prerequisite for listing, but its uniform application has been increasingly questioned, particularly in patients with acute alcohol‐associated hepatitis or rapidly progressive disease [7, 8]. Contemporary practice emphasizes individualized risk assessment that incorporates psychiatric comorbidity, social support, and engagement in addiction treatment [7, 9]. However, few models simultaneously integrate abstinence duration with structured psychosocial assessment tools such as the Stanford Integrated Psychosocial Assessment for Transplantation(SIPAT) [10, 11], socioeconomic context, and relapse severity, and the shape of the relationship between abstinence duration and relapse risk remains unclear.

We therefore aimed to evaluate how pre‐LT abstinence duration relates to the timing and severity of post‐LT alcohol relapse and to develop a multivariable risk model for relapse that incorporates abstinence, psychosocial, and socioeconomic factors.

## 2 Methods

### 2.1 Study Design

A single‐center retrospective cohort study was conducted at The Ohio State University Wexner Medical Center (OSUWMC) spanning over an 8‐year period from January 2016 through December 2023.

### 2.2 Study Population

The study cohort consisted of adult LTRs (age ≥ 18 years) who underwent primary orthotopic LT. We excluded patients who underwent LT for reasons other than ALD, those with multiple organ transplantation, re‐LT, and living donor LT. Missing data constituted 0.92% of the total dataset and were imputed using median values.

### 2.3 Data Collection

We collected comprehensive baseline characteristics including LTRs' age, sex, body mass index (BMI), race, employment status, relationship status(single, stable companion, married, divorced), education level (categorized as low, middle, or high), income level (low, middle, or high), High‐Risk Alcoholism Relapse (HRAR) score [12], pre‐LT abstinence period, participation in Alcoholics Anonymous/Alcohol and Other Drugs (AA/AOD) treatment programs [13], average daily alcohol consumption (units), history of legal issues related to alcohol (driving under the influence [DUI]), family history (1st or 2nd degree) of alcohol use disorder (AUD), the presence of failed rehabilitation attempts, tobacco use, marijuana use, psychiatric conditions, Model for End‐Stage Liver Disease‐Sodium (MELD‐Na) score at LT, SALT score [14], and SIPAT score [10, 11]. This study was conducted with approval from the institutional review board at OSUWMC (No. 2023H0392) and adhered to the principles outlined in the Declaration of Helsinki. Given the retrospective design, the requirement for informed consent was waived by the institutional review board.

### 2.4 Listing Decision Process

At our center, candidates with alcohol‐associated liver disease undergo standardized psychosocial evaluation by transplant social work and transplant psychology, incorporating structured assessment (e.g., SIPAT) and objective toxicology testing including phosphatidylethanol (PEth), with collateral verification when available. Candidates are subsequently reviewed in a multidisciplinary LT selection committee. In accordance with our institutional guideline, listing generally requires either (i) 6 months of documented abstinence or (ii) completion of ≥ 3 months of addiction treatment with ≥ 3 months of abstinence; however, for urgent high‐MELD cases, candidates deemed low relapse risk with a favorable psychosocial profile may be listed without meeting the standard duration requirements provided PEth is negative.

### 2.5 Definition of Pre‐LT Alcohol Abstinence

Pre‐LT abstinence duration was defined and measured according to our institutional protocol as the number of consecutive months patients remained alcohol‐free immediately prior to receiving LT. In accordance with OSUWMC guidelines, abstinence confirmation required either 6 months of sobriety verified by toxicology (PEth testing) and collateral reports, or a combination of 3 months of formal addiction treatment and at least 3 months of abstinence. For patients with high‐MELD scores requiring urgent listing, exceptions to the standard abstinence requirements were considered for those deemed low risk for relapse based on their psychosocial profile assessment. All patients provided documented consent for toxicology screening as part of our standard evaluation protocol.

### 2.6 Definitions of Post‐LT Alcohol Relapse and Relapse Phenotypes

Post‐LT alcohol relapse was initially identified through any detection of alcohol use, and subsequently was classified by severity into three non‐exclusive phenotypes: [1] “sustained alcohol use,” defined following Lee et al.'s criteria [14] as alcohol consumption detected on more than 100 days during the post‐LT follow‐up period; [2] “harmful drinking,” defined according to National Institute on Alcohol Abuse and Alcoholism (NIAAA) criteria [15] as consumption of > 4 drinks/day or > 14 drinks/week for men, or > 3 drinks/day or > 7 drinks/week for women, or phosphatidylethanol (PEth) levels > 200 ng/mL; and [3] “recurrent ALD,” defined as alcohol‐related liver dysfunction confirmed by blood work, imaging, clinical assessment, or liver biopsy.

Post‐LT alcohol relapse was detected through these methods: [1] patient self‐reporting during routine post‐LT follow ups (sensitivity; 88.8%) [16]; [2] monthly PEth testing as per our LT protocol (> 20 ng/mL [sensitivity; 80.9%] is set for cutoff line) [17]; [3] liver function test abnormalities; [4] collateral information from family members or caregivers; and [5] clinical assessment by transplant hepatologists. All cases of post‐LT alcohol relapse were comprehensively documented in electronic medical records and confirmed by the transplant team.

### 2.7 Post‐LT PEth Testing Protocol

Post‐LT, all ALD LTRs underwent monthly PEth testing for the first year. After the first post‐transplant year, PEth tests were serially obtained in conjunction with other laboratory tests. This systematic approach ensured regular monitoring regardless of clinical suspicion, while also allowing for additional testing when relapse was suspected.

### 2.8 Socioeconomic Status Classification

Educational attainment was categorized into a three‐tiered hierarchical classification system [18]. The “highest stratum” encompassed individuals possessing tertiary credentials including associate degrees, bachelor's degrees, or postgraduate certifications. The “intermediate stratum” comprised individuals who had matriculated at collegiate or technical institutions but had not completed their studies with formal credential acquisition. The “lowest stratum” consisted of individuals whose terminal education was confined to primary or secondary levels (high school diploma or equivalent certification).

Income estimation was performed by mapping individual patient ZIP codes to their respective counties and determining mean annual income based on the United States Census Bureau data (https://www.census.gov/), using the average income of each county [19]. Socioeconomic status was categorized into three income groups based on percentiles of the ZIP code income distribution within our sample [20]. Using the 33rd and 67th percentiles as cutoff points, patients were categorized into three groups: “Low” (< 66 987 USD, below the 33rd percentile), “Middle” (66987–87 955 USD, between the 33rd and 67th percentiles), and “High” (≥ 87 956 USD, above the 67th percentile). This classification approach reflects the relative neighborhood‐level socioeconomic status within the cohort and resulted in the following distribution: low income (*n* = 106), middle income (*n* = 85), and high income (*n* = 189). The uneven distribution across groups reflects the actual income distribution patterns within our study population.

### 2.9 Outcomes Studied

Our primary aim was to study the impact of pre‐LT abstinence period, socioeconomic factors, and psychosocial assessment on post‐LT alcohol relapse. We reported the cumulative risk by abstinence period threshold across different relapse phenotypes (sustained alcohol use, harmful drinking, and recurrent ALD). We also developed and validated multivariate prediction models for relapse risk based on these factors.

Also, we compared the performance of our models with that of the SALT score and explored a SIPAT‐free model based on routinely available clinical and socioeconomic variables to enhance potential generalizability to centers that do not routinely use formal psychosocial scoring.

### 2.10 Statistical Analysis

Continuous variables were expressed as median (interquartile range), and categorical variables as number (percentage). For between‐group comparisons, we used the Mann–Whitney U test for continuous variables and Chi‐square test or Fisher's exact test for categorical variables. To identify predictors of alcohol relapse, we conducted univariate and multivariate logistic regression analyses. Explanatory variables included patient demographics, clinical information, and psychosocial assessment scores, with odds ratios (OR) and 95% confidence intervals (CI) calculated. To address variable follow‐up duration and the competing risk of death, we performed time‐to‐event analyses for time to first post‐LT alcohol relapse. We fit a cause‐specific Cox proportional hazards model treating death prior to relapse as censoring. In addition, we estimated the 3‐year cumulative incidence of relapse using the Aalen–Johansen estimator, treating death as a competing event. Furthermore, this cohort included a small subset transplanted for acute alcohol‐associated hepatitis (AAH) and/or acute‐on‐chronic liver failure (ACLF). As sensitivity analyses, we repeated the time‐to‐event and competing‐risk analyses after excluding (i) AAH recipients and (ii) recipients with AAH and/or ACLF.

Statistical significance was set at *p* < 0.05. For threshold analysis of abstinence periods, we calculated cumulative risk by month and performed time‐dependent risk assessment using the Kaplan–Meier method. To evaluate the discriminatory ability of the prediction model, we conducted receiver operating characteristic (ROC) curve analysis and calculated the area under the curve (AUC). To validate the model's stability, we performed 5‐fold cross‐validation and reported the mean AUC. All statistical analyses were conducted using Python 3.12.4 (https://www.python.org/).

In addition to the multivariable logistic models, we calculated the SALT score for each patient according to the original definition and evaluated its discriminatory ability for any post‐LT alcohol relapse. ROC curves and corresponding AUCs were generated for the SALT score and for our SIPAT‐free multivariable model. The two ROC curves (SALT vs. SIPAT‐free model) were visually compared with illustrate incremental discrimination provided by the multivariable approach. To assess potential optimism, both the full and SIPAT‐free models were internally validated using five‐fold cross‐validation, and mean AUCs were reported.

## 3 Results

### 3.1 Baseline Characteristics

**FIGURE 1:**
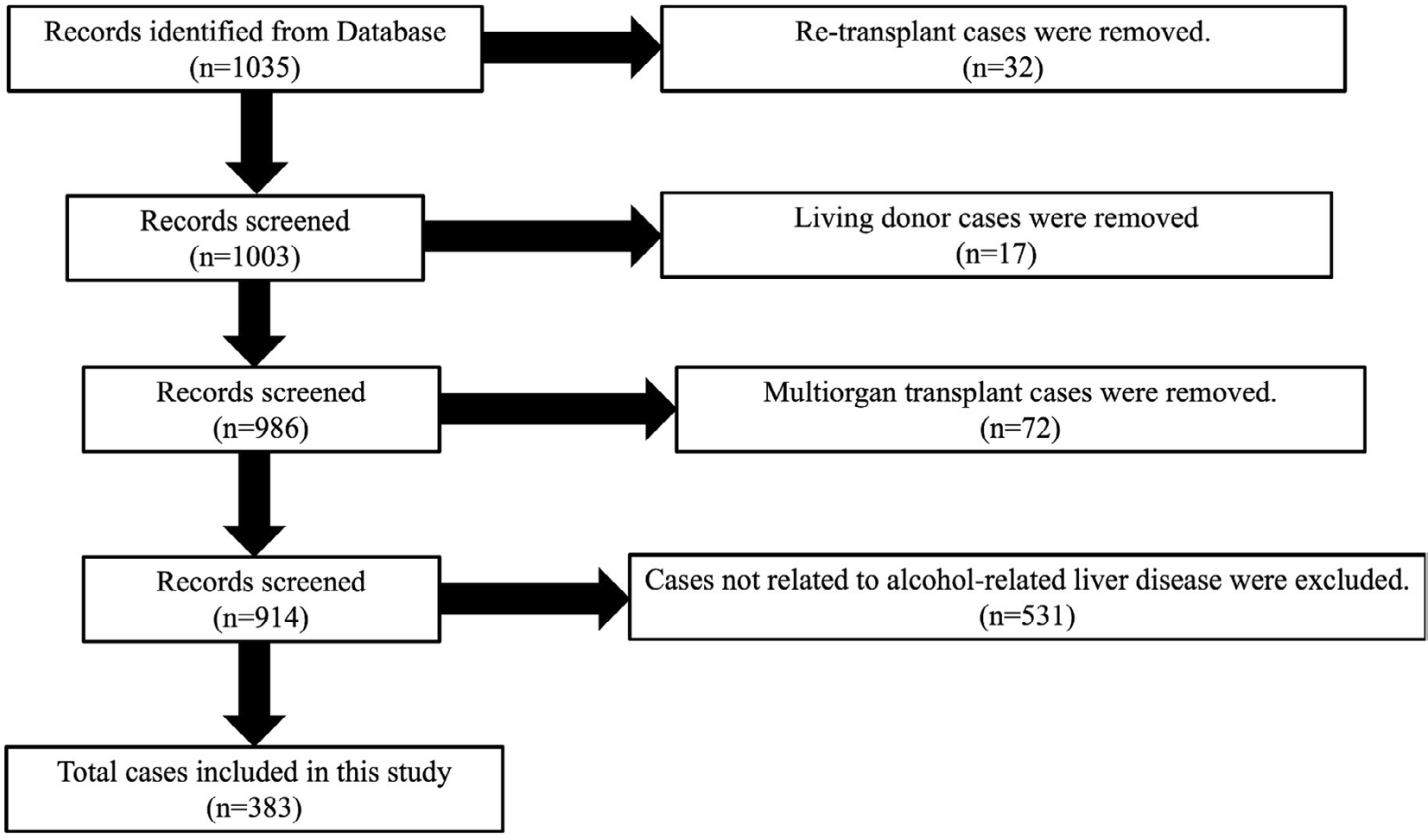
Flow chart of patient selection. Flow diagram showing the selection process of patients from the initial database of 1035 records to the final study cohort of 383 patients. Sequential exclusion of re‐transplant cases (*n* = 32), living donor cases (*n* = 17), multiorgan transplant cases (*n* = 72), and cases not related to alcohol‐related liver disease (*n* = 531) resulted in the final analytical cohort.

During the study period a total of 1035 LT were performed at OSUWMC. We excluded re‐LT (*n* = 32), living donor LT (*n* = 17), multiorgan transplants (*n* = 72), and LTRs with indications other than ALD (*n* = 531), resulting in a final cohort of 383 (37.0%) for analysis (Figure 1).

Among the 383 LTRs with ALD, total 80 (20.9%) patients experienced post‐LT alcohol relapse. These 80 relapse cases were classified into three non‐exclusive phenotypes: 62 (77.5%) met criteria for “sustained alcohol use,” 55 (68.8%) for “harmful drinking,” and 37 (46.3%) for “recurrent ALD”. The overlapping classification explains why the sum exceeds the total number of relapsed patients, as individuals could simultaneously meet criteria for multiple phenotypes. Among relapsed patients, 69 (86.2%) were ascertained via PEth testing (> 20 ng/mL), while the remaining 11 (13.8%) were ascertained via self‐reported alcohol use. Baseline clinical, psychosocial, and socioeconomic characteristics of patients meeting criteria for sustained alcohol use, harmful drinking, and recurrent ALD were broadly similar across the three relapse phenotypes (Table 2).

There were no significant differences observed between the relapse and non‐relapse groups in demographics and MELD‐Na score (Table 1). For education level, differences were observed but did not reach statistical significance (Low: 37.5% [30/80] vs. 47.5% [144/303]; Middle: 25.0% [20/80] vs. 26.7% [81/303]; High: 37.5% [30/80] vs. 25.7% [78/303], *p* = 0.10). However, income level distribution showed marginal difference (Low: 18.8% [15/80] vs. 30.0% [91/303]; Middle: 21.2% [17/80] vs. 22.4% [68/303]; High: 60.0% [48/80] vs. 46.5% [141/303], *p* = 0.07). Most importantly, LTRs in the relapse group had significantly shorter pre‐LT abstinence periods compared with the non‐relapse group (10 months [IQR: 8–14] vs. 14 months [IQR: 8–28], *p* < 0.001) and lower HRAR scores (2 [IQR: 2–3] vs. 3 [IQR: 2–4], *p* = 0.017). Significant differences were also observed in the completion rates of AA/AOD treatment programs (completed: 48.8% [39/80] vs. 31.7% [96/303], *p* < 0.001) among those relapsed and non‐relapsed. This unadjusted association should be interpreted cautiously because treatment completion is not randomized and may reflect confounding by indication. Average daily alcohol consumption pre‐LT did not differ significantly between groups (10 units [IQR: 6–17] vs. 9 units [IQR: 6–14], *p* = 0.18). Similarly, no significant differences were observed in alcohol‐related legal issues (DUI) (45.0% [36/80] vs. 43.9% [133/303], *p* = 0.10), family history (1st or 2nd degree) of AUD (21.2% [17/80] vs. 26.1% [79/303], *p* = 0.46), tobacco use (46.2% [37/80] vs. 46.2% [140/303], *p* = 1.00), marijuana use (23.8% [19/80] vs. 21.5% [65/303], *p* = 0.77), psychiatric conditions (48.8% [39/80] vs. 48.5% [147/303], *p* = 1.00), or SALT score (3 [0–6] vs. 2 [0–6], *p* = 0.46). Moreover, pre‐LT SIPAT scores tended to be higher in the relapse group, although the difference did not reach statistical significance (36 [IQR: 23–45] vs. 28 [IQR: 21–34], *p* = 0.14).

**TABLE 1:** Baseline characteristics of liver transplant recipients stratified by relapse or not.

	Relapse (*n* = 80)	Non‐relapse (*n* = 303)	Total (*n* = 383)	*p*
**Recipient**				
Age at LT, y	53 (47–59)	54 (46–61)	53 (47–60)	0.46
Sex, Male	59 (73.8)	230 (75.9)	289 (75.5)	0.80
BMI	28.3 (24.1–33.2)	28.0 (24.2–32.2)	28.1 (24.1–32.4)	0.90
MELD‐Na at LT (IQR)	24 (20–29)	24 (19–32)	24 (19–31)	0.70
Race, *n* (%)				0.21
White	71 (88.8)	282 (93.1)	353 (92.2)	
Non‐white	9 (11.2)	21 (6.9)	30 (7.8)	
Employed	17 (21.2)	73 (24.1)	90 (23.5)	0.69
Married/Stable companion	47 (58.8)	191 (63.0)	238 (62.1)	0.31
Education level				0.10
Low	30 (37.5)	144 (47.5)	174 (45.4)	
Middle	20 (25.0)	81 (26.7)	108 (28.2)	
High	30 (37.5)	78 (25.7)	101 (26.4)	
Income level				
Low	15 (18.8)	91 (30.0)	106 (27.7)	0.07
Middle	17 (21.2)	68 (22.4)	85 (22.2)	
High	48 (60.0)	141 (46.5)	189 (49.3)	
HRAR score	2 (2–3)	3 (2–4)	2 (2–3)	0.017
Period of abstinence prior to LT, month, (IQR)	10 (8–14)	14 (8–28)	13 (8–24)	< 0.001
Degree of AA/AOD completion pre‐LT				< 0.001
Completed	39 (48.8)	96 (31.7)	135 (35.2)	
Not completed	41 (51.2)	207 (68.3)	248 (64.8)	
Average units/day of alcohol, *n* (IQR)	10 (6–17)	9 (6–14)	9 (6–15)	0.18
AUD legal issue (DUI), *n*(%)	36 (45.0)	133 (43.9)	177 (46.2)	0.10
Family Hx of AUD (1st or 2nd degree), *n*(%)	17 (21.2)	79 (26.1)	96 (25.1)	0.46
Hx of pharm Tx of AUD, *n*(%)	9 (11.2)	27 (8.91)	36 (9.4)	0.52
Failed rehab attempts, *n*(%)	20 (25.0)	38 (12.5)	58 (15.1)	0.006
Tobacco use, *n*(%)	37 (46.2)	140 (46.2)	177 (46.2)	1.00
Marijuana use, *n*(%)	19 (23.8)	65 (21.5)	84 (21.9)	0.77
Psychiatric condition, *n*(%)	39 (48.8)	147 (48.5)	186 (48.6)	1.00
Pre‐LT SIPAT score, (IQR)	36 (23–45)	28 (21–34)	29 (21–37)	0.14
SALT score, (IQR)	3 (0–6)	2 (0–6)	2 (0–6)	0.46

*Note:* Continuous variables: median [IQR]; categorical variable: number (%).

Abbreviations: AA, alcoholics anonymous; AOD, alcohol and other drugs; AUD, alcohol use disorder; BMI, body mass index; DUI, driving under the influence; HRAR, high‐risk alcoholism relapse score; IQR, interquartile range; LT, liver transplant; MELD‐Na, model for end‐stage liver disease‐sodium; SALT, sustained alcohol use post‐liver transplant; SIPAT, Stanford Integrated Psychosocial Assessment for Transplantation.

**TABLE 2:** Baseline characteristics of liver transplant recipients stratified by types of recurrence.

	Sustained use (*n* = 62)	Harmful drink (*n* = 55)	Recurrent ALD (*n* = 37)
**Recipient**			
Age at LT, y	53 (46–59)	52 (46–59)	51 (43–56)
Sex, Male	51(82.3)	42 (76.4)	29 (78.4)
BMI	29.0 (24.5–33.4)	28.1 (23.1–33.3)	28.5 (23.4–33.1)
MELD‐Na at LT (IQR)	24 (20–30)	25 (20–31)	24 (21–29)
Race, *n* (%)			
White	54 (87.1)	49 (89.1)	35 (94.6)
Non‐white	8 (12.9)	6 (10.9)	2 (5.4)
Employed	15 (24.2)	11 (20.0)	10 (27.0)
Married/Stable companion	38 (61.2)	31 (56.4)	20 (54.1)
Education level			
Low	24 (38.7)	21 (38.2)	12 (32.4)
Middle	15 (24.2)	16 (29.1)	9 (24.3)
High	23 (37.1)	18 (32.7)	16 (43.2)
Income level			
Low	6 (9.7)	6 (10.9)	3 (8.1)
Middle	15 (24.2)	14 (25.5)	10 (27.0)
High	41 (66.1)	35 (63.6)	24 (64.9)
HRAR score, *n* (%)	3 (2–3)	3 (2–4)	3 (2–4)
Period of abstinence prior to LT, month, (IQR)	10 (7–14)	9 (7–14)	9 (6–15)
Degree of AA/AOD completion pre‐LT			
Completed	28 (45.2)	29 (52.7)	14 (37.8)
Not completed	34 (54.8)	10 (47.3)	23 (62.2)
Average units/day of alcohol, *n* (IQR)	10 (6–17)	11 (6–20)	10 (6–17)
AUD legal issue (DUI), *n*(%)	33 (53.2)	31 (56.4)	16 (43.2)
Family Hx of AUD (1st or 2nd degree), *n*(%)	14 (22.6)	14 (25.5)	7 (18.9)
Tobacco use, *n*(%)	28 (45.2)	25 (45.5)	16 (43.2)
Marijuana use, *n*(%)	16 (25.8)	13 (23.6)	5 (13.5)
Psychiatric condition, *n*(%)	29 (46.8)	24 (43.6)	16 (43.2)
Pre‐LT SIPAT score, (IQR)	36 (23–45)	36 (23–46)	30 (23–39)

*Note:* Continuous variables: median [IQR]; categorical variable: number (%).

Abbreviations: AA, alcoholics anonymous; AOD, alcohol and other drugs; AUD, alcohol use disorder; BMI, body mass index; DUI, driving under the influence; HRAR, high‐risk alcoholism relapse score; IQR, interquartile range; LT, liver transplant; MELD‐Na, model for end‐stage liver disease‐sodium; SIPAT, Stanford Integrated Psychosocial Assessment for Transplantation.

**TABLE 3:** Multivariate and univariate analyses for alcohol relapse.

	Univariate			Multivariate		
	OR	95% Cl	*p*	OR	95% Cl	*p*
**Recipient**						
Age at LT	0.99	0.97–1.02	0.44	1.02	0.98–1.05	0.32
Sex, male	0.89	0.51–1.57	0.69	0.90	0.47–1.75	0.75
BMI	1.00	0.95–1.04	0.89	1.02	0.97–1.07	0.48
MELD‐Na at LT	0.99	0.97–1.02	0.63	0.97	0.93–1.01	0.08
Race, white	0.59	0.26–1.34	0.20	0.60	0.22–1.60	0.31
Employed	0.84	0.46–1.52	0.55	0.79	0.39–1.60	0.52
Single, divorced, widowed	0.64	0.35–1.20	0.17	1.13	0.63–2.04	0.68
Education level						
Low	0.54	0.30–0.96	0.04	0.44	0.22–0.90	0.02
Middle	0.64	0.34–1.22	0.18	0.55	0.26–1.14	0.11
High	Reference			Reference		
Income level						
Low	0.31	0.15–0.64	0.002	0.62	0.31–1.23	0.17
Middle	0.75	0.41–1.39	0.36	0.87	0.41–1.88	0.73
High	Reference			Reference		
HRAR score	1.42	1.12–1.80	0.004	1.05	0.72–1.53	0.80
Period of abstinence prior to LT, month,	0.97	0.94–0.98	0.002	0.96	0.93–0.99	0.004
Completion of AA/AOD pre‐LT	0.85	0.39–1.85	0.68	0.20	0.01–4.40	0.31
Average units/day of alcohol	1.04	1.01–1.08	0.009	1.04	0.99–1.10	0.12
AUD legal issue (DUI)	1.56	0.95–2.56	0.08	1.36	0.75–2.47	0.31
Family Hx of AUD (1st or 2nd degree)	0.77	0.42–1.39	0.38	0.55	0.27–1.10	0.09
Failed rehab attempt	2.40	1.30–4.42	0.005	1.96	0.90–4.24	0.09
Tobacco use	1.00	0.61–1.64	0.99	0.92	0.50–1.70	0.80
Marijuana use	1.14	0.63–2.04	0.66	0.94	0.48–1.85	0.86
Psychiatric condition	1.01	0.62–1.65	0.97	0.71	0.39–1.30	0.27
Pre‐LT SIPAT score	1.04	1.02–1.06	< 0.001	1.03	1.01–1.06	0.01
SALT score	1.04	0.95–1.15	0.40			

*Note:* Values are odds ratios (OR) and 95% confidence intervals (CI) from multivariable logistic regression.

Abbreviations: AA, alcoholics anonymous; AOD, alcohol and other drugs; AUD, alcohol use disorder; BMI, body mass index; DUI, driving under the influence; HRAR, high‐risk alcoholism relapse score; IQR, interquartile range; LT, liver transplant; MELD‐Na, model for end‐stage liver disease‐sodium; SALT, sustained alcohol use post‐liver transplant; SIPAT, Stanford Integrated Psychosocial Assessment for Transplantation.

### 3.2 Predictors of Alcohol Relapse

Table 3 presents the results of univariate and multivariate analyses. On univariate analysis, pre‐LT abstinence period (OR: 0.97; CI: 0.94–0.98, *p* = 0.002), HRAR score (OR: 1.42; CI: 1.12–1.80, *p* = 0.004), average alcohol consumption (OR: 1.04; CI: 1.01–1.08, *p* = 0.009), pre‐LT SIPAT score (OR: 1.04; CI: 1.02–1.06, *p* < 0.001), low income level (OR: 0.31; CI: 0.15–0.64, *p* = 0.002), and low education level (OR: 0.54; CI: 0.30–0.96, *p* = 0.04) were significantly associated with post‐LT alcohol relapse. In the full multivariable model including all candidate covariates, pre‐LT abstinence period (OR: 0.96; 95% CI 0.93–0.99, *p* = 0.004), pre‐LT SIPAT score (OR: 1.03; 95% CI 1.01–1.06, *p* = 0.01), and low education level (OR: 0.44; 95% CI 0.22–0.90, *p* = 0.02) were identified as independent predictors of relapse. Notably, each additional month of abstinence was associated with a 4% reduction in the odds of relapse, while each one‐point increase in SIPAT score conferred a 3% increase in relapse odds. These three variables therefore formed the basis of the simplified prediction model described below.

**FIGURE 2:**
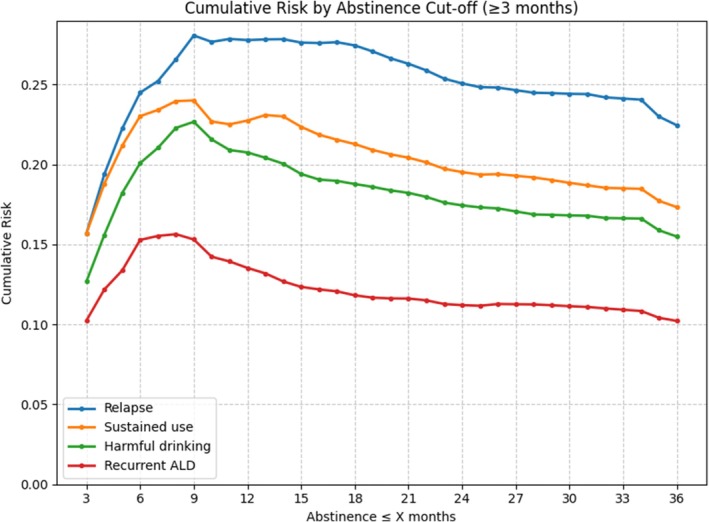
Cumulative risk by abstinence period across relapse phenotypes. Time‐dependent changes in cumulative risk for any post‐LT alcohol relapse (blue line), sustained alcohol use (orange line), harmful drinking (green line), and recurrent ALD (red line) stratified by pre‐LT abstinence duration. All phenotypes showed peak risk around 9 months of abstinence, followed by a gradual decline over time, suggesting a non‐linear relationship between abstinence duration and relapse risk.

Cumulative risk analysis by abstinence period threshold, as illustrated in Figure 2, demonstrated time‐dependent changes in the risk of any post‐LT alcohol relapse (blue line), sustained alcohol use (orange line), harmful drinking (green line), and recurrent ALD (red line). Most importantly, all relapse phenotypes showed peak risk at around 9 months of abstinence, followed by a gradual decline over time. This non‐linear relationship suggests that the commonly applied “Six‐month abstinence rule” may not optimally stratify relapse risk.

### 3.3 Predictive Model Performance

**FIGURE 3:**
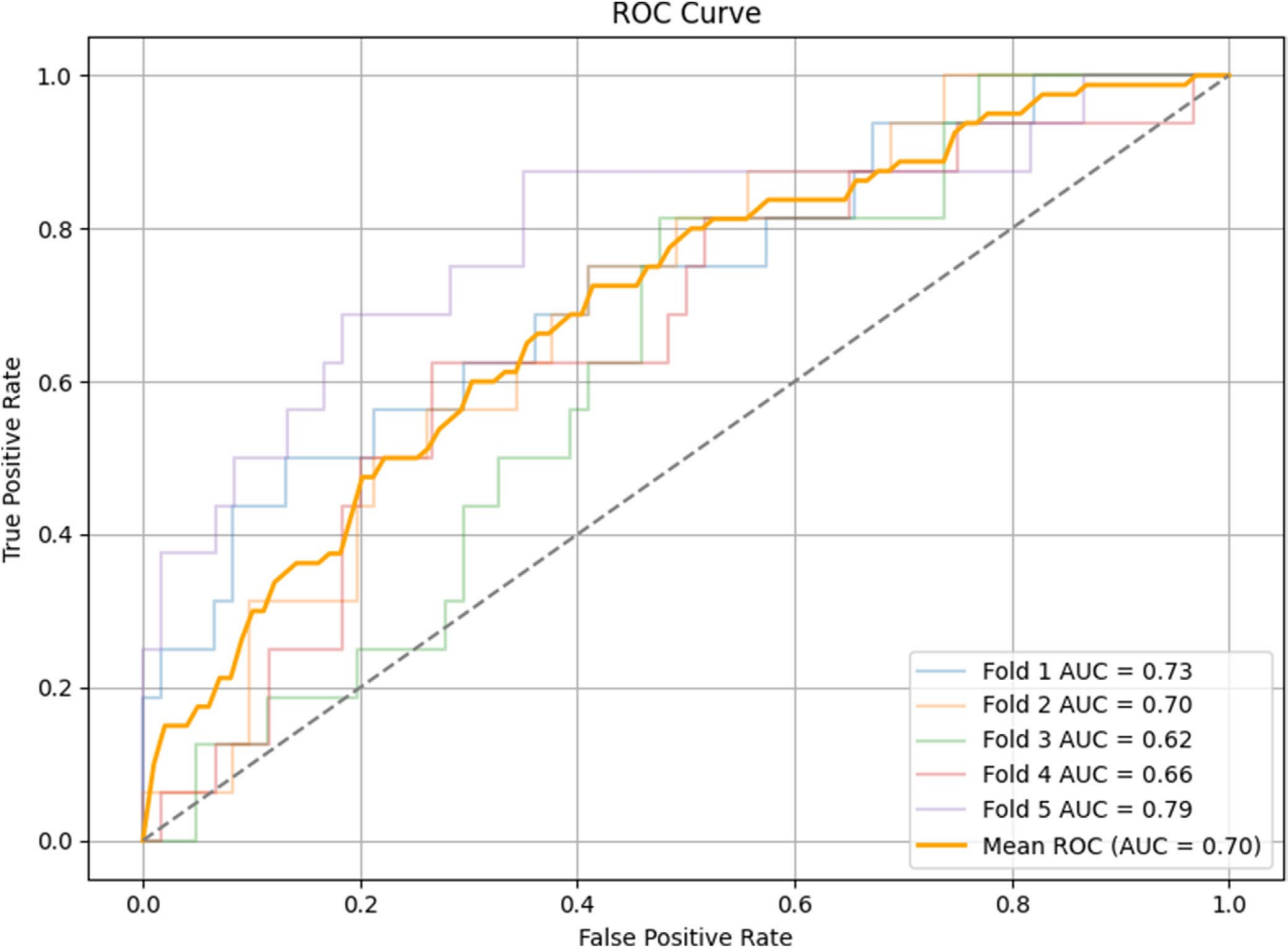
ROC curve analysis of the prediction model. Receiver operating characteristic (ROC) curve showing the performance of the prediction model incorporating pre‐LT abstinence period, SIPAT score, and education level. The area under the curve (AUC) of 0.70 indicates moderate predictive accuracy. Internal validation using 5‐fold cross‐validation demonstrated consistent performance across folds (Fold 1: AUC = 0.73, Fold 2: AUC = 0.70, Fold 3: AUC = 0.62, Fold 4: AUC = 0.66, Fold 5: AUC = 0.79), with a mean AUC of 0.70.

Using these three predictors, we then constructed a simplified multivariable model for relapse risk. This reduced model yielded the following predictive equation for post‐LT alcohol relapse: logit(*p*) = −1.53−0.034 × (abstinence period prior to LT in months) + 0.037 × (Pre‐LT SIPAT score) − 0.60 × (Education = Low) − 0.56 × (Education = Middle). In this equation, Education = High was the reference category (0). The probability of post‐LT alcohol relapse can be calculated using the standard logistic transformation: *p* = 1/[1 + exp.(−logit(*p*))]. The ROC curve analysis (Figure 3) of prediction model yielded an AUC of 0.70, indicating moderate predictive accuracy. Internal validation using 5‐fold cross‐validation demonstrated generally consistent performance across folds (Fold 1: AUC = 0.73, Fold 2: AUC = 0.70, Fold 3: AUC = 0.62, Fold 4: AUC = 0.66, Fold 5: AUC = 0.79), with a mean AUC of 0.70. Importantly, these results suggest that the combination of pre‐LT abstinence period, SIPAT score, and education level provides a stable and clinically useful prediction of post‐LT alcohol relapse.

**FIGURE 4:**
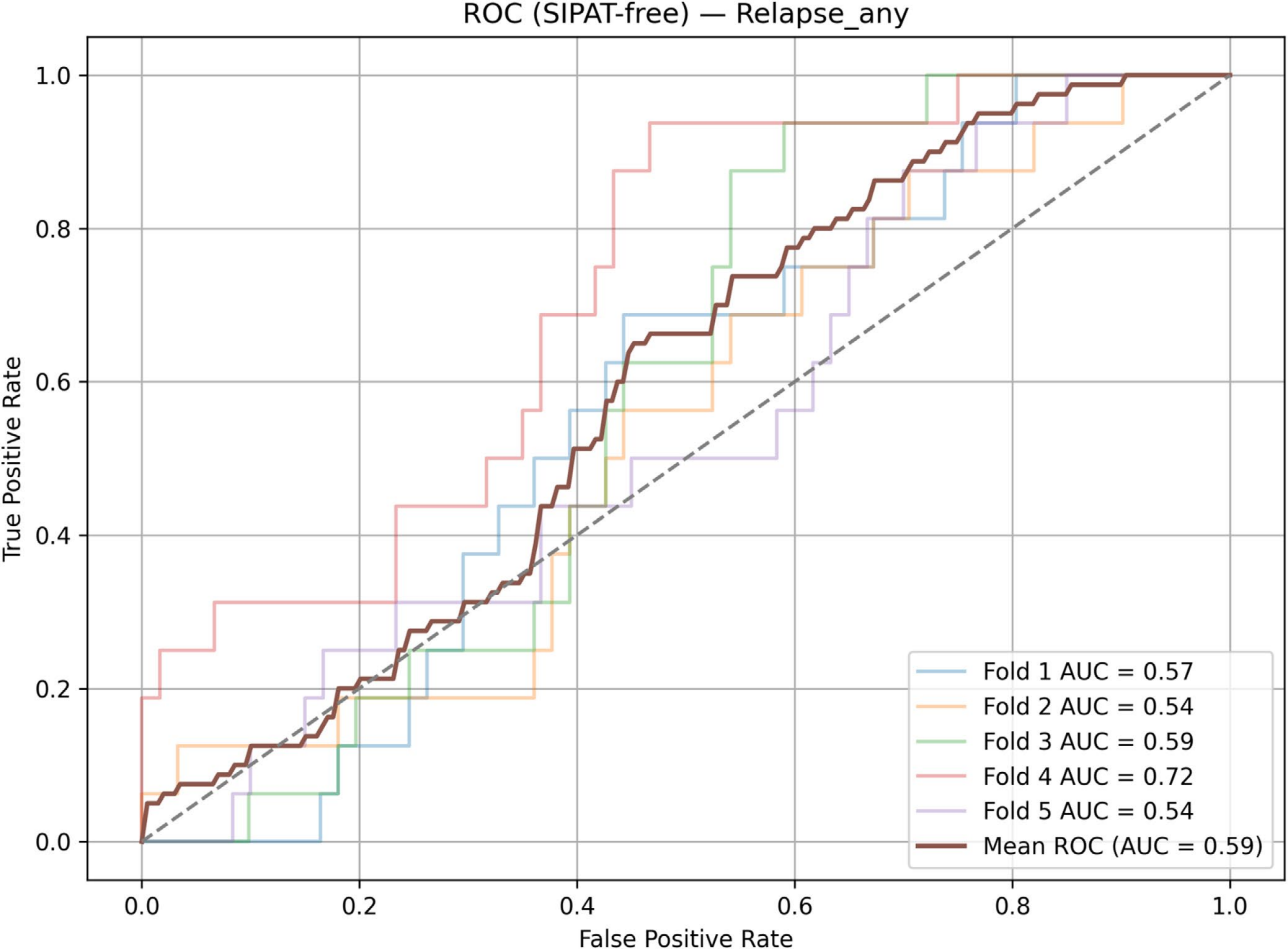
ROC curves for the SIPAT‐free multivariable model predicting any post‐LT. Colored lines represent each of the five cross‐validation folds, and the dark line indicates the mean ROC curve (mean AUC = 0.59).

**FIGURE 5:**
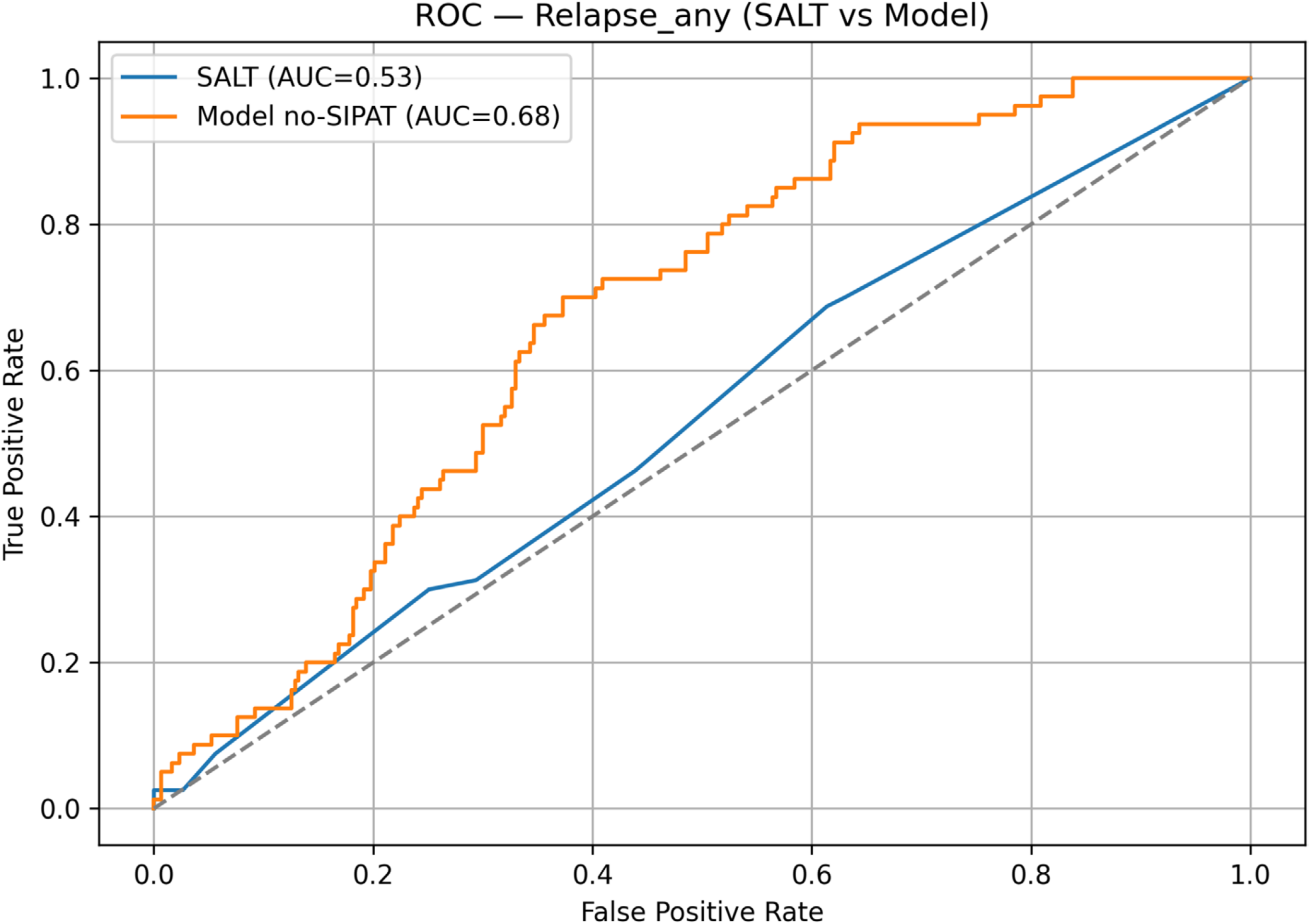
Comparison of ROC curves for the SALT score and the SIPAT‐free multivariable model predicting any post‐LT alcohol relapse. The SALT score showed poor discrimination (AUC = 0.53), whereas the SIPAT‐free model demonstrated better discrimination (AUC = 0.68).

The multivariable SIPAT‐free model that included only clinical, behavioral, and socioeconomic variables showed fair discrimination for any post‐LT alcohol relapse. The apparent AUC of the SIPAT‐free model was 0.68, whereas the mean AUC from five‐fold cross‐validation was 0.59, indicating some optimism but preserved ranking ability across folds (Figure 4). When the SALT score was evaluated in our cohort, it showed poor discrimination for any post‐LT alcohol relapse, with an AUC of 0.53. In contrast, the SIPAT‐free multivariable model demonstrated better discrimination, with its ROC curve lying above that of SALT across most probability thresholds (Figure 5).

In complementary time‐to‐event analyses, the cause‐specific Cox model for time to first any post‐LT alcohol relapse (treating death prior to relapse as censoring) showed findings consistent with the primary logistic regression (Table S1). When accounting for death as a competing event, the 3‐year cumulative incidence of any post‐LT alcohol relapse was 19.8%, and was higher in the SIPAT‐high group than in the SIPAT‐low group (23.0% vs. 16.7%; median split at 29) (Figure S2).

In sensitivity analyses excluding AAH recipients (*n* = 362), the adjusted cause‐specific Cox results and the 3‐year cumulative incidence estimates were materially unchanged (Table S2; Figure S3). Findings were also robust when excluding recipients with AAH and/or ACLF (*n* = 357) (Table S3; Figure S4), suggesting that the observed associations were not driven by acute/rapidly progressive presentations.

## 4 Discussion

We comprehensively analyzed factors associated with post‐LT alcohol relapse in LTRs for ALD. Among the 383 patients analyzed, 80 LTRs (20.9%) experienced post‐LT alcohol relapse, and multivariate analysis identified pre‐LT abstinence period, SIPAT score, and low education level as independent predictors. The predictive model combining these factors demonstrated moderate accuracy with an AUC of 0.70, and its stability was confirmed through 5‐fold cross‐validation. Furthermore, cumulative risk analysis of abstinence periods revealed a common non‐linear pattern across all relapse phenotypes—“sustained alcohol use,” “harmful drinking,” and “recurrent ALD”—where relapse risk peaked at approximately 9 months and subsequently decreased over time. These results suggest that regardless of the post‐LT alcohol relapse phenotypes, the relationship between abstinence period and relapse risk exhibits similar time‐dependent variations.

ALD is a leading indication for LT [1], and post‐LT alcohol relapse is associated with serious outcomes, including graft dysfunction [3] and patient mortality [4]. With reported relapse rates of approximately 20%–25% over a 4‐year post‐LT period [5], accurate relapse risk assessment is critically important for the effective utilization of limited organ resources [6]. The conventional “Six‐month abstinence rule” has been criticized as overly uniform, and recent approaches emphasize the importance of individualized risk assessment incorporating psychiatric evaluation and social support factors [7, 9]. This standardized approach is particularly challenging to apply in urgent cases such as acute alcoholic hepatitis [7, 8]. While various factors including SIPAT, socioeconomic background, and AUD severity have been implicated in relapse risk, the development of integrated prediction models incorporating these factors remains in its early stages. The present study aimed to elucidate the relationship between abstinence period and cumulative risk thresholds, as well as associations between socioeconomic factors and relapse risk, to develop a more accurate and clinically useful risk assessment model.

The multivariate analysis in this study identified pre‐LT abstinence period, SIPAT score, and low education level as independent predictors of post‐LT alcohol relapse. These findings indicate that alcohol relapse is influenced by complex interactions between psychosocial and socioeconomic factors rather than by a single determinant. The significance of the SIPAT score aligns with studies by de Gottardi et al. [21] and Donnadieu‐Rigole et al. [22], and is consistent with Chuncharunee et al.'s meta‐analysis [5] demonstrating an association between psychiatric comorbidity and increased relapse risk. The SIPAT score is a standardized measure that comprehensively evaluates transplant candidates' psychological stability, social support, substance use history, and treatment compliance [10, 11]. Our study revealed a quantitative relationship where each one‐point increase in SIPAT score conferred a 3% increase in relapse risk, supporting the clinical importance of pre‐LT psychosocial assessment not merely as a screening tool but as a predictor of post‐LT outcomes. This is consistent with Pfitzmann et al.'s findings on “lack of social support” [23] and Chuncharunee et al.'s identification of “complex psychiatric illness” as predictive factors for relapse [5]. Notably, low education level was associated with decreased relapse risk. This counterintuitive finding has recently been corroborated by Meinders et al. [24], who found that patients with higher education levels (undergraduate or postgraduate) had a significantly higher risk for post‐LT alcohol relapse compared with those with primary or secondary education. Several explanations might account for this seemingly paradoxical result. First, a potential selection bias may exist; patients with lower education levels may have undergone more stringent selection processes to be deemed suitable candidates for LT [25]. Second, heightened perception of social stigma among those with lower educational attainment may enhance internal motivation for sustained abstinence [26]. Additionally, according to Binswanger's investigation [27], medical teams tend to provide intentionally strengthened support to potentially high‐risk groups, which may contribute to relapse prevention. However, education likely serves as a proxy for broader socioeconomic and behavioral factors, and this association should not be interpreted as causal. Residual confounding, center‐specific selection practices, and potential differences in relapse ascertainment across socioeconomic strata may have influenced this finding; therefore, it should be considered hypothesis‐generating and warrants external validation.

Analysis of the relationship between abstinence period and relapse risk also yielded clinically significant insights. The cumulative risk analysis by abstinence period threshold illustrated in Figure 2 revealed a non‐linear relationship in which relapse risk peaked at approximately 9 months of abstinence and subsequently decreased gradually over time across all phenotypes—any post‐LT alcohol relapse, sustained alcohol use, harmful drinking, and recurrent ALD. A notable aspect of this study is the classification of relapse into three distinct phenotypes: “sustained alcohol use (> 100 days),” “harmful drinking (based on NIAAA criteria),” and “recurrent ALD,” with individual assessment of each phenotype's association with abstinence period. Previous studies have predominantly treated post‐LT alcohol relapse as a single phenotype, and to our knowledge, no reports have examined the relationship between abstinence period and different relapse severity levels. The observation of similar time‐dependent risk variations regardless of relapse phenotype provides a novel perspective on the clinical significance of abstinence periods.

These observations challenge the traditional 6‐month abstinence rule as an optimal risk stratification tool. The highest relapse risk in our cohort appears to extend beyond 6 months, and patients who maintain abstinence beyond approximately 9 months may represent a subgroup with greater capacity to sustain behavior change. These findings are consistent with prior reports showing acceptable outcomes in patients transplanted with shorter periods of abstinence and no consistent association between a rigid 6‐month threshold and long‐term outcomes [8]. Together with our non‐linear risk pattern, this highlights the importance of considering qualitative aspects of abstinence—such as engagement with treatment and psychosocial stability—rather than duration alone when assessing transplant candidacy and planning post‐LT addiction care [28]. In parallel, we developed a multivariable prediction model that integrates abstinence duration, SIPAT score, and education level. This model showed moderate discrimination for any post‐LT alcohol relapse (AUC 0.70), with consistent performance on five‐fold cross‐validation. A simpler SIPAT‐free model based on routinely available clinical and socioeconomic variables achieved only modest discrimination (apparent AUC 0.68; mean cross‐validated AUC 0.59) but still outperformed the SALT score in our cohort (AUC 0.53). These findings suggest that non‐psychosocial variables alone are insufficient for high‐accuracy prediction, but that combining them with abstinence duration in a multivariable framework yields incremental value over existing tools. At the same time, the superior performance of the full model including SIPAT underscores the added prognostic information provided by structured psychosocial assessment. Importantly, our intent is not for these models to serve as rigid listing gatekeepers, but rather as tools to stratify post‐LT alcohol relapse risk and to guide the intensity and structure of follow‐up—such as the frequency of clinic visits, use of PEth monitoring, and early referral to addiction medicine or counseling services—for patients who already meet center‐specific candidacy criteria. Importantly, pre‐LT AA/AOD completion and failed rehabilitation attempts are baseline characteristics and do not evaluate the effectiveness of post‐LT risk‐adapted follow‐up strategies, which require prospective testing.

Our study has several limitations. First, as a single‐center retrospective cohort study, selection and measurement biases may exist. However, the relatively large sample size of 383 patients and internal validation through 5‐fold cross‐validation provide a reasonable degree of reliability and generalizability for our findings. The higher AA/AOD completion rate among patients who later relapsed likely reflects confounding by indication, as individuals judged to be at higher relapse risk may be preferentially referred to and more intensively engaged in structured AA/AOD programs. Therefore, unadjusted group differences in completion rates do not necessarily indicate lack of benefit or harm. Our observational design limits causal inference regarding the effect of AA/AOD completion on relapse. Although our primary modeling used logistic regression, we additionally conducted time‐to‐event sensitivity analyses to account for variable follow‐up duration and the competing risk of death. A cause‐specific Cox model (death censored) and a competing‐risk approach using the Aalen–Johansen estimator yielded findings consistent with the primary analysis (Table S1 and Figure S2). Consistent results in these sensitivity cohorts suggest that our primary associations are unlikely to be driven by acute/rapidly progressive presentations (Tables S2 and S3; Figures S3 and S4). Nevertheless, external validation of the developed model has not been conducted, making the confirmation of predictive performance in different patient populations and healthcare environments an important area for future research. External validation through multi‐center collaborative studies with broader geographical and demographic backgrounds would further enhance the model's generalizability. Second, relapse assessment was primarily based on self‐reporting and information from family members, raising the possibility of underreporting. However, this limitation was mitigated by incorporating objective indicators such as liver function tests and blood alcohol markers, including PEth. This methodological limitation has been similarly noted in studies by Young et al. [29] and Pageaux et al. [30] and represents a common challenge in post‐LT alcohol relapse research. Third, our income level classification was based on ZIP code mapping to county‐level census data rather than individual income assessment, which may not accurately reflect patients' actual financial circumstances. Fourth, standardized criteria for defining abstinence periods and socioeconomic factors have not been established. Nevertheless, the classification methods employed in this study are consistent with previous research, ensuring interpretability of the results. Future development of more objective and standardized assessment methods is desirable.

In conclusion, our study demonstrated that pre‐LT abstinence period, SIPAT score, and education level are independent predictors of relapse following LT for ALD. Particularly regarding abstinence periods, we revealed a non‐linear relationship where relapse risk peaked at approximately 9 months and subsequently decreased. The novelty of this study lies in its classification of relapse into three severity phenotypes—“sustained alcohol use,” “harmful drinking,” and “recurrent ALD”—and its individual assessment of each phenotype's association with abstinence period. The predictive model combining these independent factors demonstrated moderate predictive accuracy (AUC 0.70), suggesting the importance of a comprehensive approach incorporating psychosocial assessment and socioeconomic factors beyond simple abstinence periods in evaluating transplant candidacy for patients with ALD. To enhance clinical translation, we provide a proposed implementation framework integrating abstinence duration, psychosocial risk (SIPAT), and socioeconomic context (education as an available proxy) to support multidisciplinary decision‐making for transplant evaluation and to tailor the intensity of post‐transplant monitoring and addiction‐focused follow‐up (Figure S1). Future studies should focus on external validation of this predictive model, verification through prospective research, and evaluation of the effectiveness of intervention strategies based on the model.

## Author Contributions

**Ayato Obana:** conceptualization, investigation, funding acquisition, writing – original draft, methodology, validation, visualization, writing – review and editing, formal analysis, data curation, project administration, software, resources. **Garren Montgomery:** writing – review and editing, data curation. **Miho Akabane:** writing – review and editing. **Matthew Hamilton:** writing – review and editing. **Navdeep Singh:** writing – review and editing. **Musab Alebrahim:** writing – review and editing. **Austin Schenk:** writing – review and editing. **Sylvester Black:** writing – review and editing. **Kenneth Washburn:** writing – review and editing. **Khalid Mumtaz:** writing – review and editing, supervision.

## Funding

This work was supported by Uehara Memorial Foundation, 202540084.

## Disclosure

Declaration of Generative AI and AI‐Assisted Technologies in the Writing Process: No generative AI or AI‐assisted technologies were used in the writing process of this manuscript.

## Ethics Statement

This study was conducted with approval from the institutional review board at OSUWMC (No. 2023H0392) and adhered to the principles outlined in the Declaration of Helsinki. Given the retrospective design, the requirement for informed consent was waived by the institutional review board.

## Conflicts of Interest

The authors declare no conflicts of interest.

## Supporting information


**Figure S1:** Proposed management strategy integrating abstinence duration, psychosocial risk, and socioeconomic context to guide transplant evaluation and post‐LT follow‐up in alcohol‐associated liver disease. Flow chart illustrating a proposed implementation framework that integrates pre‐transplant abstinence duration (months), psychosocial risk assessed by SIPAT, and socioeconomic context (education as a proxy in this study) to support relapse risk stratification (low, intermediate, high) and to tailor the intensity of post‐transplant monitoring and addiction‐focused follow‐up. The figure also depicts consideration of expedited listing for urgent high‐MELD candidates with a favorable profile and negative PEth, alongside the standard listing pathway when feasible. This framework is intended to facilitate multidisciplinary decision‐making and should be prospectively validated.

**Figure S2:** Three‐year cumulative incidence of alcohol relapse with death as a competing risk in the overall cohort (*n* = 383). Cumulative incidence functions were estimated using the Aalen–Johansen estimator and are shown overall and stratified by the pre‐transplant SIPAT score dichotomized at the cohort median (median = 29). SIPAT, Stanford Integrated Psychosocial Assessment for Transplantation.

**Figure S3:** Three‐year cumulative incidence of alcohol relapse with death as a competing risk after excluding recipients transplanted for acute alcohol‐associated hepatitis (AAH) (*n* = 362). Cumulative incidence functions were estimated using the Aalen–Johansen estimator and are shown overall and stratified by the pre‐transplant SIPAT score dichotomized at the cohort median (median = 28). SIPAT, Stanford Integrated Psychosocial Assessment for Transplantation.

**Figure S4:** Three‐year cumulative incidence of alcohol relapse with death as a competing risk after excluding recipients with acute alcohol‐associated hepatitis (AAH) and/or acute‐on‐chronic liver failure (ACLF) (*n* = 357). Cumulative incidence functions were estimated using the Aalen–Johansen estimator and are shown overall and stratified by the pre‐transplant SIPAT score dichotomized at the cohort median (median = 28). SIPAT, Stanford Integrated Psychosocial Assessment for Transplantation.

**Table S1:** Cause‐specific Cox proportional hazards model for time to first post‐LT alcohol relapse (any alcohol use); death prior to relapse was treated as censoring (*n* = 383).
**Table S2:** Cause‐specific Cox proportional hazards model for time to first post‐LT alcohol relapse (any alcohol use); death prior to relapse was treated as censoring (excluding AAH cohort [*n* = 362]).
**Table S3:** Cause‐specific Cox proportional hazards model for time to first post‐LT alcohol relapse (any alcohol use); death prior to relapse was treated as censoring (excluding AAH, ACLF cohort [*n* = 357]).

## Data Availability

The datasets generated and analyzed during the current study are available from the corresponding author upon reasonable request.
