# Supplementary figures and images for: Multifactor Risk Stratification for Post‐Transplant Alcohol Relapse Using Abstinence, Psychosocial, and Socioeconomic Factors
Source: Ann Gastroenterol Surg. 2026 Feb 15;10(4):1217–30. doi: 10.1002/ags3.70193 (PMC13327090; doi:10.1002/ags3.70193)

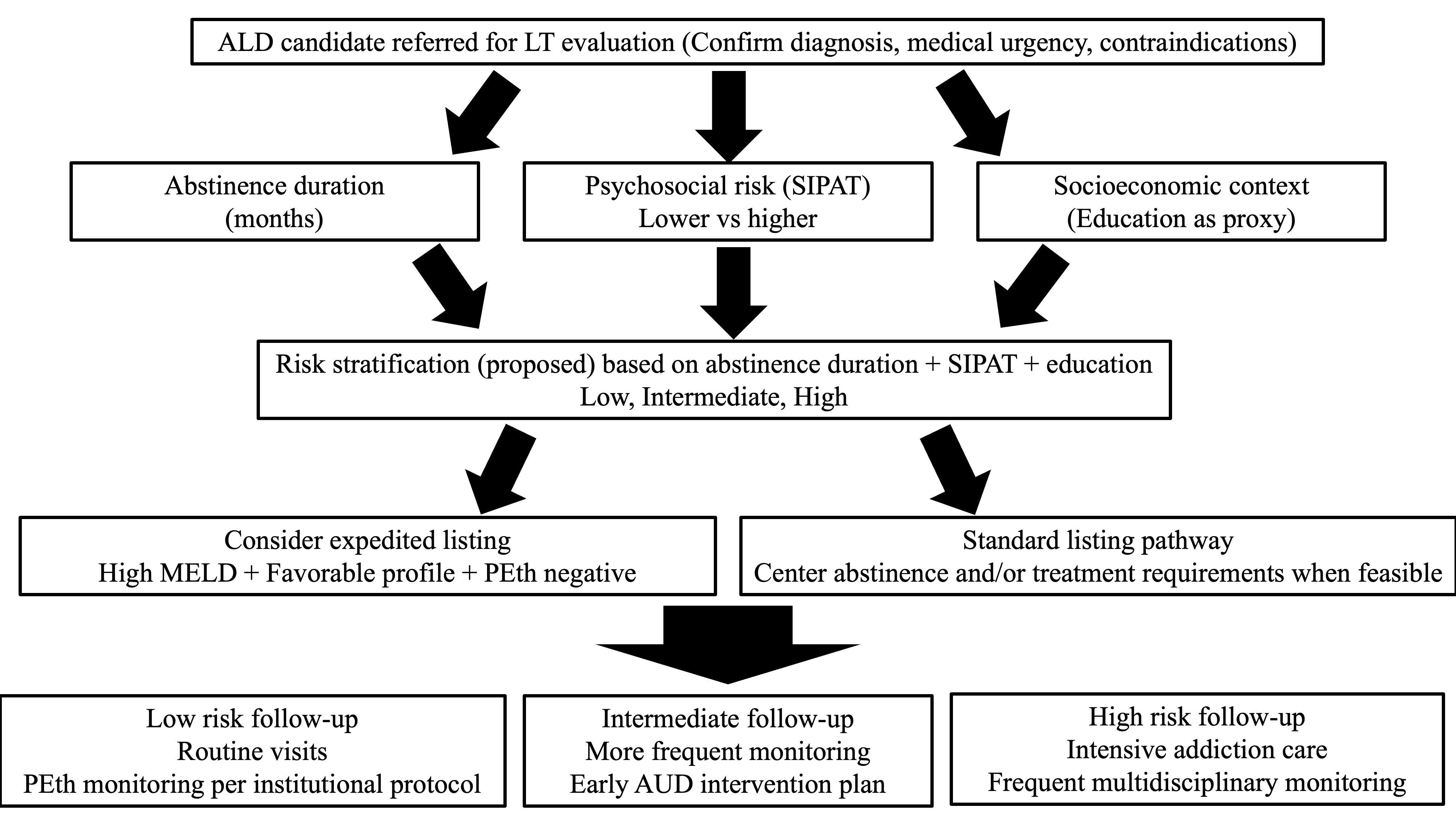

Supplement: Supplementary file 1 — Figure S1: Proposed management strategy integrating abstinence duration, psychosocial risk, and socioeconomic context to guide transplant evaluation and post‐LT follow‐up in alcohol‐associated liver disease. Flow chart illustrating a proposed implementation framework that integrates pre‐transplant abstinence duration (months), psychosocial risk assessed by SIPAT, and socioeconomic context (education as a proxy in this study) to support relapse risk stratification (low, intermediate, high) and to tailor the intensity of post‐transplant monitoring and addiction‐focused follow‐up. The figure also depicts consideration of expedited listing for urgent high‐MELD candidates with a favorable profile and negative PEth, alongside the standard listing pathway when feasible. This framework is intended to facilitate multidisciplinary decision‐making and should be prospectively validated. [file AGS3-10-1217-s002.tif]

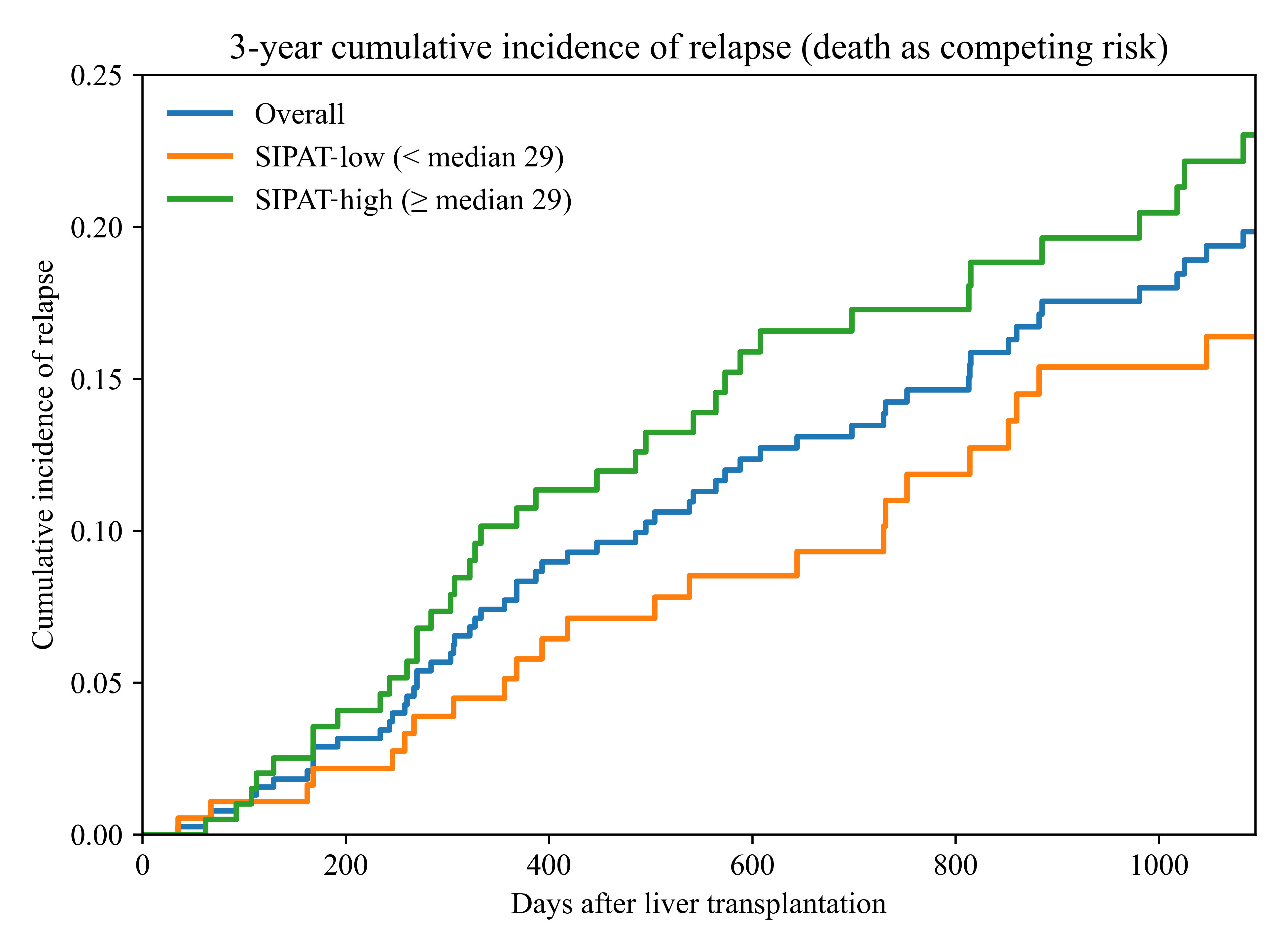

Supplement: Supplementary file 2 — Figure S2: Three‐year cumulative incidence of alcohol relapse with death as a competing risk in the overall cohort (n = 383). Cumulative incidence functions were estimated using the Aalen–Johansen estimator and are shown overall and stratified by the pre‐transplant SIPAT score dichotomized at the cohort median (median = 29). SIPAT, Stanford Integrated Psychosocial Assessment for Transplantation. [file AGS3-10-1217-s003.tif]

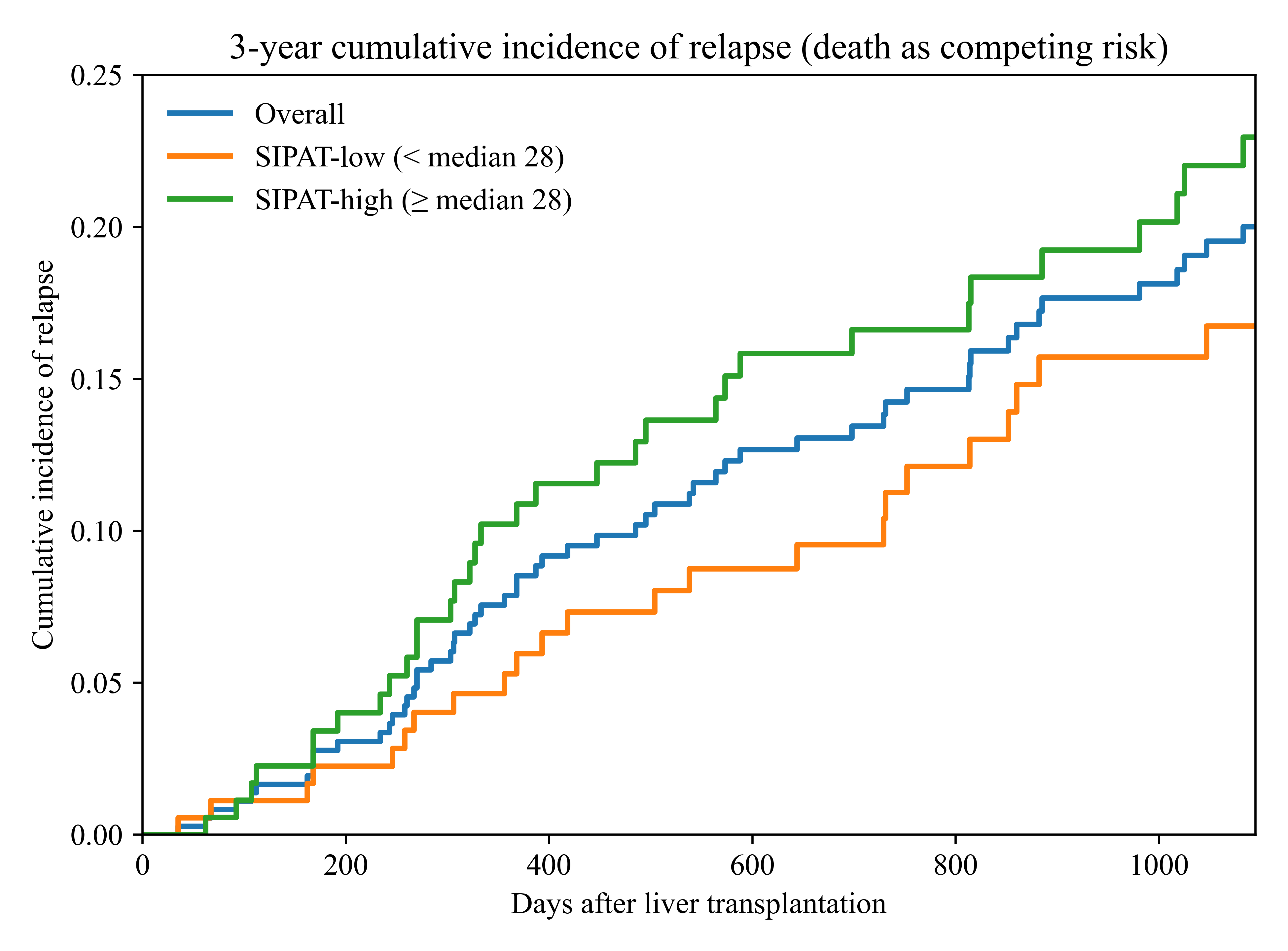

Supplement: Supplementary file 3 — Figure S3: Three‐year cumulative incidence of alcohol relapse with death as a competing risk after excluding recipients transplanted for acute alcohol‐associated hepatitis (AAH) (n = 362). Cumulative incidence functions were estimated using the Aalen–Johansen estimator and are shown overall and stratified by the pre‐transplant SIPAT score dichotomized at the cohort median (median = 28). SIPAT, Stanford Integrated Psychosocial Assessment for Transplantation. [file AGS3-10-1217-s005.tif]

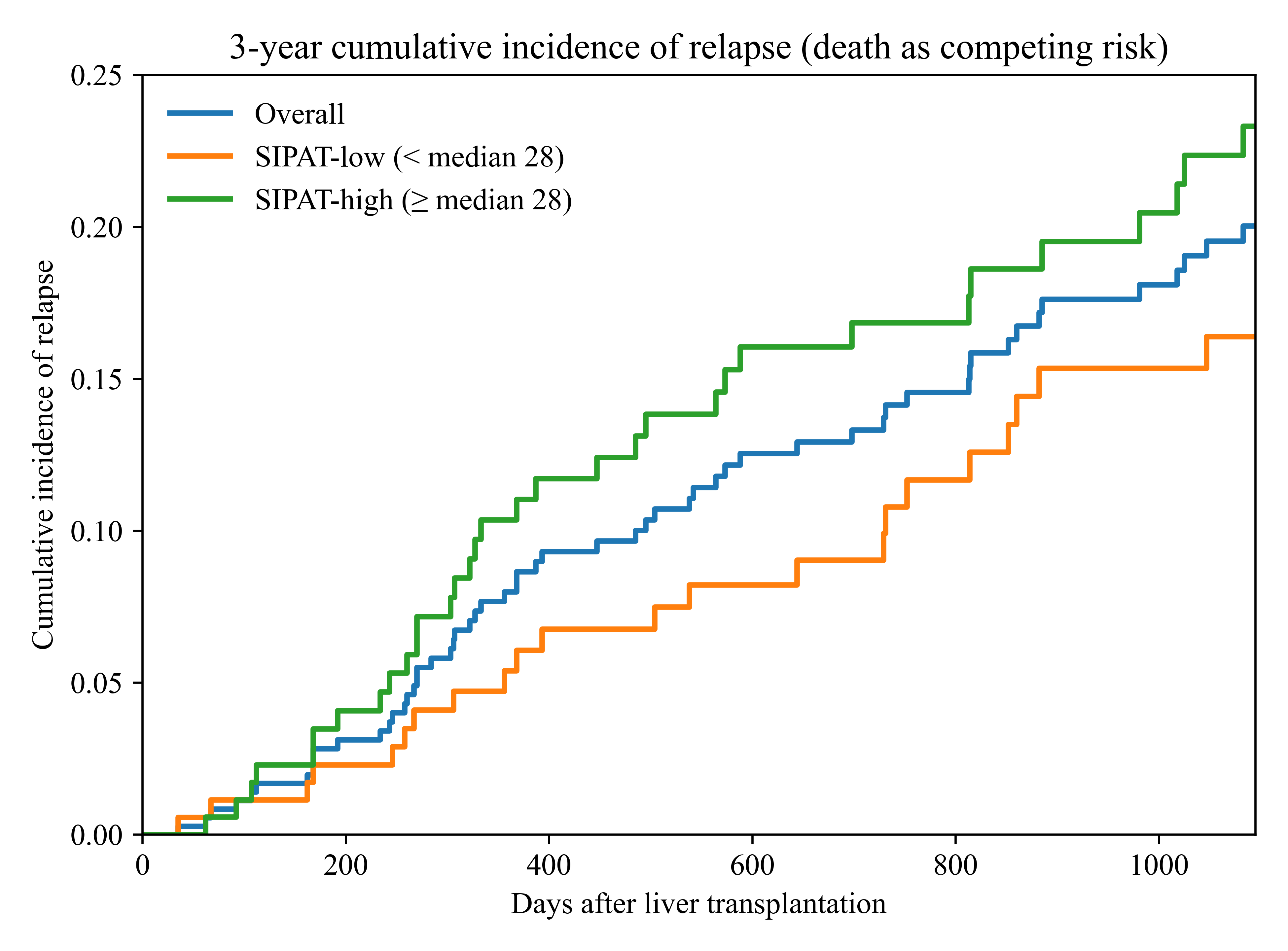

Supplement: Supplementary file 4 — Figure S4: Three‐year cumulative incidence of alcohol relapse with death as a competing risk after excluding recipients with acute alcohol‐associated hepatitis (AAH) and/or acute‐on‐chronic liver failure (ACLF) (n = 357). Cumulative incidence functions were estimated using the Aalen–Johansen estimator and are shown overall and stratified by the pre‐transplant SIPAT score dichotomized at the cohort median (median = 28). SIPAT, Stanford Integrated Psychosocial Assessment for Transplantation. [file AGS3-10-1217-s004.tif]
