# Supplementary material for: Multifactor Risk Stratification for Post‐Transplant Alcohol Relapse Using Abstinence, Psychosocial, and Socioeconomic Factors
Source: Ann Gastroenterol Surg. 2026 Feb 15;10(4):1217–30. doi: 10.1002/ags3.70193 (PMC13327090; doi:10.1002/ags3.70193)
Supplement: Supplementary file 5 — Table S1: Cause‐specific Cox proportional hazards model for time to first post‐LT alcohol relapse (any alcohol use); death prior to relapse was treated as censoring (n = 383). Table S2: Cause‐specific Cox proportional hazards model for time to first post‐LT alcohol relapse (any alcohol use); death prior to relapse was treated as censoring (excluding AAH cohort [n = 362]). Table S3: Cause‐specific Cox proportional hazards model for time to first post‐LT alcohol relapse (any alcohol use); death prior to relapse was treated as censoring (excluding AAH, ACLF cohort [n = 357]). [file AGS3-10-1217-s001.docx]

Supplementary Table 1. Cause-specific Cox proportional hazards model for time to first post-LT alcohol relapse (any alcohol use); death prior to relapse was treated as censoring. (n=383)

|  | **Univariate** |  |  | **Multivariate** |  |  |
| --- | --- | --- | --- | --- | --- | --- |
|  | **HR** | **95%Cl** | **p** | **HR** | **95%Cl** | **p** |
| **Recipient** |  |  |  |  |  |  |
| Age at LT | 0.98 | 0.96–1.01 | 0.14 | 1.00 | 0.98–1.02 | 0.66 |
| Sex, Male | 0.79 | 0.48–1.30 | 0.35 | 0.85 | 0.55–1.32 | 0.48 |
| BMI | 1.00 | 0.96–1.04 | 0.92 | 1.01 | 0.97–1.04 | 0.67 |
| MELD-Na at LT | 0.99 | 0.96–1.01 | 0.34 | 0.99 | 0.97–1.01 | 0.30 |
| Race, White | 0.61 | 0.30–1.21 | 0.16 | 0.71 | 0.37–1.35 | 0.29 |
| Employed | 1.07 | 0.62–1.83 | 0.81 | 0.99 | 0.63–1.56 | 0.98 |
| Single, divorced, widowed | 1.20 | 0.77–1.88 | 0.42 | 1.10 | 0.75–1.61 | 0.63 |
| Education level |  |  |  |  |  |  |
| Low | 0.61 | 0.39–0.96 | 0.03 | 0.65 | 0.43–0.99 | 0.04 |
| Middle | 1.08 | 0.65–1.80 | 0.77 | 0.91 | 0.58–1.43 | 0.69 |
| High | Reference |  |  | Reference |  |  |
| Income level |  |  |  |  |  |  |
| Low | 0.61 | 0.36–1.03 | 0.06 | 0.74 | 0.48–1.13 | 0.16 |
| Middle | 0.85 | 0.48–1.52 | 0.59 | 0.88 | 0.54–1.43 | 0.61 |
| High | Reference |  |  | Reference |  |  |
| HRAR score | 1.30 | 1.06–1.61 | 0.01 | 1.05 | 0.86–1.29 | 0.61 |
| Period of abstinence prior to LT, month, | 0.97 | 0.95–0.98 | 0.001 | 0.99 | 0.98–0.99 | 0.02 |
| Completion of AA/AOD pre-LT | 1.88 | 1.21–2.93 | 0.004 | 1.36 | 0.92–2.01 | 0.12 |
| Average units/day of alcohol | 1.04 | 1.02–1.08 | 0.001 | 1.03 | 0.99–1.06 | 0.06 |
| AUD legal issue (DUI) | 1.55 | 0.99–2.41 | 0.052 | 1.22 | 0.83–1.78 | 0.31 |
| Family Hx of AUD (1^st^ or 2^nd^ degree) | 0.76 | 0.44–1.30 | 0.31 | 0.71 | 0.45–1.11 | 0.13 |
| Failed rehab attempt | 1.70 | 1.02–2.82 | 0.04 | 1.22 | 0.75–1.99 | 0.42 |
| Tobacco use | 1.04 | 0.67–1.62 | 0.85 | 0.92 | 0.63–1.36 | 0.68 |
| Marijuana use | 1.35 | 0.81–2.27 | 0.25 | 1.13 | 0.72–1.79 | 0.59 |
| Psychiatric condition | 1.01 | 0.65–1.57 | 0.96 | 0.84 | 0.57–1.23 | 0.38 |
| Pre-LT SIPAT score | 1.02 | 1.01–1.04 | 0.003 | 1.01 | 1.00–1.03 | 0.10 |

Hazard ratios (HRs) and 95% confidence intervals (CIs) from a cause-specific Cox proportional hazards model for time to relapse; death was censored.

*Abbreviations.* LT: Liver transplant, BMI: Body mass index, MELD-Na: Model for End-Stage Liver Disease-Sodium, IQR: Interquartile range, HRAR: High Risk Alcoholism Relapse score, AA: Alcoholics Anonymous, AOD: Alcohol and Other Drugs, AUD: Alcohol Use Disorder, DUI: Driving Under the Influence, SIPAT: Stanford Integrated Psychosocial Assessment for Transplantation, SALT: Sustained Alcohol use Post-Liver Transplant

Supplementary Table 2. Cause-specific Cox proportional hazards model for time to first post-LT alcohol relapse (any alcohol use); death prior to relapse was treated as censoring. (excluding AAH cohort [n=362])

|  | **Univariate** |  |  | **Multivariate** |  |  |
| --- | --- | --- | --- | --- | --- | --- |
|  | **HR** | **95%Cl** | **p** | **HR** | **95%Cl** | **p** |
| **Recipient** |  |  |  |  |  |  |
| Age at LT | 0.98 | 0.96–1.01 | 0.11 | 0.99 | 0.97–1.01 | 0.52 |
| Sex, Male | 0.75 | 0.45–1.25 | 0.27 | 0.85 | 0.55–1.33 | 0.49 |
| BMI | 0.99 | 0.95–1.03 | 0.73 | 1.00 | 0.97–1.04 | 0.87 |
| MELD-Na at LT | 0.98 | 0.96–1.01 | 0.19 | 0.98 | 0.96–1.01 | 0.17 |
| Race, White | 0.60 | 0.30–1.21 | 0.16 | 0.73 | 0.38–1.39 | 0.33 |
| Employed | 1.08 | 0.62–1.88 | 0.79 | 0.98 | 0.62–1.56 | 0.94 |
| Single, divorced, widowed | 1.11 | 0.70–1.75 | 0.66 | 1.04 | 0.70–1.54 | 0.85 |
| Education level |  |  |  |  |  |  |
| Low | 0.61 | 0.39–0.97 | 0.04 | 0.68 | 0.44–1.03 | 0.07 |
| Middle | 1.22 | 0.73–2.04 | 0.45 | 1.02 | 0.64–1.62 | 0.94 |
| High | Reference |  |  | Reference |  |  |
| Income level |  |  |  |  |  |  |
| Low | 0.59 | 0.35–1.02 | 0.06 | 0.71 | 0.46–1.09 | 0.11 |
| Middle | 0.74 | 0.40–1.37 | 0.34 | 0.81 | 0.49–1.33 | 0.40 |
| High | Reference |  |  | Reference |  |  |
| HRAR score | 1.32 | 1.06–1.63 | 0.01 | 1.06 | 0.86–1.31 | 0.59 |
| Period of abstinence prior to LT, month, | 0.96 | 0.94–0.99 | 0.001 | 0.99 | 0.98–0.99 | 0.02 |
| Completion of AA/AOD pre-LT | 1.92 | 1.22–3.01 | 0.004 | 1.32 | 0.89–1.97 | 0.17 |
| Average units/day of alcohol | 1.05 | 1.02–1.09 | 0.001 | 1.03 | 0.99–1.06 | 0.06 |
| AUD legal issue (DUI) | 1.55 | 0.99–2.43 | 0.058 | 1.22 | 0.82–1.80 | 0.34 |
| Family Hx of AUD (1^st^ or 2^nd^ degree) | 0.78 | 0.45–1.34 | 0.37 | 0.71 | 0.45–1.12 | 0.14 |
| Failed rehab attempt | 1.65 | 0.98–2.78 | 0.057 | 1.17 | 0.71–1.92 | 0.53 |
| Tobacco use | 1.04 | 0.66–1.63 | 0.87 | 0.91 | 0.61–1.36 | 0.65 |
| Marijuana use | 1.36 | 0.80–2.32 | 0.25 | 1.12 | 0.70–1.80 | 0.63 |
| Psychiatric condition | 1.03 | 0.65–1.61 | 0.91 | 0.85 | 0.58–1.26 | 0.42 |
| Pre-LT SIPAT score | 1.02 | 1.01–1.04 | 0.004 | 1.01 | 1.00–1.03 | 0.13 |

NOTE: Hazard ratios (HR) from Cox proportional hazards models

*Abbreviations.* LT: Liver transplant, BMI: Body mass index, MELD-Na: Model for End-Stage Liver Disease-Sodium, IQR: Interquartile range, HRAR: High Risk Alcoholism Relapse score, AA: Alcoholics Anonymous, AOD: Alcohol and Other Drugs, AUD: Alcohol Use Disorder, DUI: Driving Under the Influence, SIPAT: Stanford Integrated Psychosocial Assessment for Transplantation

Supplementary Table 3. Cause-specific Cox proportional hazards model for time to first post-LT alcohol relapse (any alcohol use); death prior to relapse was treated as censoring. (excluding AAH, ACLF cohort [n=357])

|  | **Univariate** |  |  | **Multivariate** |  |  |
| --- | --- | --- | --- | --- | --- | --- |
|  | **HR** | **95%Cl** | **p** | **HR** | **95%Cl** | **p** |
| **Recipient** |  |  |  |  |  |  |
| Age at LT | 0.98 | 0.96–1.01 | 0.08 | 0.99 | 0.97–1.01 | 0.45 |
| Sex, Male | 0.71 | 0.42–1.18 | 0.18 | 0.82 | 0.52–1.28 | 0.37 |
| BMI | 1.00 | 0.96–1.04 | 0.89 | 1.00 | 0.97–1.04 | 0.76 |
| MELD-Na at LT | 0.98 | 0.96–1.01 | 0.18 | 0.98 | 0.96–1.01 | 0.19 |
| Race, White | 0.59 | 0.29–1.18 | 0.14 | 0.71 | 0.37–1.36 | 0.30 |
| Employed | 1.11 | 0.64–1.95 | 0.70 | 1.01 | 0.63–1.61 | 0.97 |
| Single, divorced, widowed | 1.15 | 0.73–1.84 | 0.55 | 1.07 | 0.72–1.59 | 0.74 |
| Education level |  |  |  |  |  |  |
| Low | 0.63 | 0.39–1.00 | 0.05 | 0.70 | 0.46–1.07 | 0.10 |
| Middle | 1.26 | 0.75–2.12 | 0.38 | 1.05 | 0.66–1.67 | 0.85 |
| High | Reference |  |  | Reference |  |  |
| Income level |  |  |  |  |  |  |
| Low | 0.63 | 0.37–1.09 | 0.10 | 0.74 | 0.48–1.15 | 0.18 |
| Middle | 1.26 | 0.75–2.12 | 0.38 | 0.83 | 0.50–1.37 | 0.46 |
| High | Reference |  |  | Reference |  |  |
| HRAR score | 1.30 | 1.05–1.63 | 0.02 | 1.06 | 0.86–1.32 | 0.58 |
| Period of abstinence prior to LT, month, | 0.96 | 0.94–0.99 | 0.001 | 0.99 | 0.98–0.99 | 0.02 |
| Completion of AA/AOD pre-LT | 2.00 | 1.26–3.17 | 0.003 | 1.36 | 0.90–2.05 | 0.14 |
| Average units/day of alcohol | 1.05 | 1.02–1.09 | 0.002 | 1.03 | 0.99–1.06 | 0.08 |
| AUD legal issue (DUI) | 1.51 | 0.96–2.40 | 0.08 | 1.20 | 0.81–1.79 | 0.36 |
| Family Hx of AUD (1^st^ or 2^nd^ degree) | 0.70 | 0.40–1.25 | 0.23 | 0.68 | 0.42–1.08 | 0.11 |
| Failed rehab attempt | 1.58 | 0.92–2.72 | 0.10 | 1.14 | 0.68–1.91 | 0.62 |
| Tobacco use | 1.07 | 0.68–1.70 | 0.77 | 0.94 | 0.63–1.41 | 0.77 |
| Marijuana use | 1.34 | 0.78–2.31 | 0.30 | 1.11 | 0.69–1.80 | 0.66 |
| Psychiatric condition | 1.06 | 0.67–1.67 | 0.81 | 0.87 | 0.58–1.29 | 0.48 |
| Pre-LT SIPAT score | 1.02 | 1.01–1.04 | 0.006 | 1.01 | 1.00–1.03 | 0.16 |

NOTE: Hazard ratios (HR) from Cox proportional hazards models

*Abbreviations.* LT: Liver transplant, BMI: Body mass index, MELD-Na: Model for End-Stage Liver Disease-Sodium, IQR: Interquartile range, HRAR: High Risk Alcoholism Relapse score, AA: Alcoholics Anonymous, AOD: Alcohol and Other Drugs, AUD: Alcohol Use Disorder, DUI: Driving Under the Influence, SIPAT: Stanford Integrated Psychosocial Assessment for Transplantation
